# Promoting dual careers at higher education institutions: 31 benefits ranked by the project Student Athletes Erasmus+ Mobility in Europe (SAMEurope)

**DOI:** 10.3389/fspor.2024.1407194

**Published:** 2024-07-01

**Authors:** Carlos Hernando Domingo, Marta Renau Michavila, Per Thorén, Johan Bankel, Magnus Karlsteen, Sami Kalaja, Minna Rasinaho, Aki Karjalainen, Swantje Scharenberg, Pascale Kohler, Florian Agneray, Alexia Deflon, Dorothée Brac de la Perriere, María Pilar Marín Gil

**Affiliations:** ^1^ Sports Service, Department of Education and Specific Didactics, Universitat Jaume I, Castellón, Spain; ^2^ Sports Service, Department of Translation and Communication, Universitat Jaume I, Castellón, Spain; ^3^ Education Management Support, Chalmers University of Technology, Gothenburg, Sweden; ^4^ Department of Physics, Chalmers University of Technology, Gothenburg, Sweden; ^5^ Faculty of Sport and Health Sciences, University of Jyväskylä, Jyväskylä, Finland; ^6^ Institute of Sports and Sports Science, Karlsruhe Institute of Technology, Karlsruhe, Germany; ^7^ International Affairs, Karlsruhe Institute of Technology, Karlsruhe, Germany; ^8^ Sport Centre, Institut national des sciences Appliquées de Lyon, Villeurbanne, France; ^9^ European and International Relations Office, Institut National des Sciences Appliquées de Lyon, Villeurbanne, France; ^10^ Sports Service, Universitat Jaume I, Castellón, Spain

**Keywords:** dual career, benefits, universities, elite sport, athletes, SAMEurope

## Abstract

**Introduction:**

The project Student Athletes Erasmus+ Mobility in Europe identified and defined a total of 31 benefits offered to dual-career student athletes who are combining their university studies with high-level training. The project was co-funded by the European Union and carried out by five universities: Chalmers University of Technology (Sweden), Institut national des sciences appliquées de Lyon (France), University of Jyväskylä (Finland), Karlsruhe Institute of Technology (Germany), and Universitat Jaume I (Spain).

**Methods:**

The purpose of the study was to rank these benefits by combining the perspectives of the university staff and the student athletes from each university in the consortium. The university staff included experts from sports services and the international relations office. A questionnaire was also sent to the dual-career athletes enrolled at the consortium's universities. Of the 514 dual-career athletes, 208 (116 women) completed the questionnaire. The overall response rate was 40.47%. The university staff assessed the importance of each benefit, how easy it was to implement at the institution, and whether or not the university offered the benefit to its students. The dual-career students rated each of the 31 benefits and indicated whether or not they had used them. A specific methodology was designed to rank these benefits using the ratings of the university staff and the student athletes. Intra-group and inter-group Pearson correlations were performed.

**Results:**

The results show a strong and significant correlation between the benefits from the perspective of the university staff (*r* = 0.710, *p* = 7.76E-7) and from the perspective of the students (*r* = 0.715, *p* = 2.44E-6). The correlation is moderate and significant when the benefits are correlated from the perspective of the two groups as a whole (0.363, *p* = 0.045), with the three most important benefits being the free use of sports facilities, justification for absences, and the adaptation of the pace of studies.

**Discussion:**

The study makes visible the commitment of higher education institutions to facilitating the dual career of student athletes and identifies those benefits that may be of greater interest to European universities as a whole. The European perspective has been considered, while respecting the specificities of each university and the country in which it is located.

## 1 Introduction

The development of the term “dual career”, which emerged in 2007 as part of a resolution of the European Commission (1), focuses on the need to combine one's studies or professional life with the development of a sporting career [EU (2, 3)]. Since 2012, the European Commission's guidelines have highlighted developments in this area and the importance of addressing these two aspects for elite student athletes.

Thus, the European Commission recognizes and implements measures to mitigate the impact of this double profile for elite athletes. In particular, several publications show the complexity and current barriers to combining higher education with a sporting career (4–7). Stress and demands on student athletes have been identified as key factors impacting the development of dual careers, which has led to an investigation into the psychological issues affecting this group of students (8–10).

Another aspect is the need to take into account the limited duration of a sporting career (11). A good balance between academics and training helps facilitate more effectively the transition of student athletes to the professional market (12, 13).

Aquilina and Henry identified as early as 2010 that there were differences in the national organizational structures that serve elite student athletes. This helped highlight the fact that cultural and organizational conditions and contexts are elements that promote, to a greater or lesser extent, the development of the dual careers of student athletes (14).

These circumstances, together with the impetus provided by the European Commission through the creation of the Erasmus+ program in 2014 and an increase in funding (11,235,479.19 €), promote the development of projects in this field and partnerships for their implementation (15).

The guidelines of the European Commission and the increased funding have led to studies and publications that focus on the development of dual careers between 2015 and 2021 (16). Different approaches are used in the development of the projects, which help to clarify the current situation. Some focus on assessing the needs of the students through qualitative surveys in focus groups (17) or surveys of the university population (18–21). Others take into account the opinion of dual-career experts at the university (22); European Education and Culture Executive Agency (23).

Given all these conditions, one key element needed to contextualize this study is to determine which definition of dual-career athlete is to be analyzed within the dual-career framework. First, we looked at elite athletes who were combining their sporting career with their studies at an institution of higher education (24, 25). We also analyzed the concept of an “elite athlete”, a concept that is still somewhat confusing and requires further definition, as interpretations are not homogeneous (26, 27). The European Commission considers an elite athlete to be an athlete who represents his or her country in his or her sport (26). Li and Sum (7), however, define an elite athlete as someone who has competed at the national, international, or Olympic level, or is a professional athlete or player (7). Thus, it is necessary to clarify this aspect in order to determine precisely which student athletes are being addressed. This paper defines student athletes as those who are taking part in a dual-career program at one of the universities participating in the study.

Thus, the project Student Athletes Erasmus+ Mobility in Europe (SAMEurope, project 101050378) of the Erasmus+ program was created in 2022 with the aim of developing the recommendations of the European Commission and the EU Guidelines on Dual Careers of Athletes (2), paying particular attention to Guidelines 28 and 29 which focus directly and specifically on the mobility of students between the different countries of the European Union and the creation of transnational consortia which have as *a priori*ty high-level university student athletes.

The SAMEurope project, co-funded by the European Commission, consists of a consortium of five European universities: Chalmers University of Technology (Sweden), Institut national des sciences appliquées de Lyon (France), University of Jyväskylä (Finland), Karlsruhe Institute of Technology (Germany) and Universitat Jaume I (Spain). The consortium is involved in several measures aimed at promoting the mobility of high-level student athletes. One of the first measures is to identify the programs and benefits offered to and used by students pursuing dual careers at these universities. The knowledge of these measures can help different higher education institutions to propose similar lines of action in order to homogenise the development of the dual career in higher education institutions in the European Union.

Thus, this paper aims to: (i) identify which benefits are offered to dual-career students at the universities in the consortium; (ii) rank these benefits at the consortium level, taking into account the perspectives of the university staff and the student athletes; (iii) establish intragroup and intergroup relationships to analyze and define improvement measures to facilitate the development of dual careers. Our hypothesis is that the ranking of benefits presented by the students is not related to the ranking presented by the university staff of the consortium.

## 2 Materials and methods

### 2.1 Subjects

The study is set within the context of the five universities participating in the SAMEurope project:

Institut National des Sciences Appliquées de Lyon (France)
Karlsruhe Institute of Technology (Germany)
Chalmers University of Technology (Sweden)
University of Jyväskylä (Finland)
Universitat Jaume I de Castellón (Spain)

Two distinct groups participated in the study: university staff with at least 5 years of experience in dual-career programs or international mobility at the consortium's universities and dual-career students at these five universities.

### 2.2 Procedures

The study was divided into three phases and was approved by all of the partner universities and by the Ethics Committee of the Universitat Jaume I of Castellón (CEISH/27/2022). It complies with the criteria laid down in the Declaration of Helsinki.

The first phase of the study was carried out from September 30, 2022 to October 20, 2022 and consisted of five semi-structured qualitative interviews with sports experts in charge of dual-career athletes at each of the consortium's universities. The purpose of the interview was to gain insight into the status of dual-career programs at the five universities, located in different countries and contexts. The aim was to draw parallels between the programs in order to obtain an overview in Europe of dual careers at the consortium universities.

The interviews were accompanied by a questionnaire with six blocks of open-ended questions (see Supplementary Material S1):

1. Description of the elite athlete program
2. Access to the program
3. Academic benefits of the dual career
4. Sports benefits of the dual career
5. Other benefits related to a dual career
6. Relationship with student athletes

Each interview lasted approximately 90–120 min. The information obtained from the interviews was analyzed, taking into account the similarities and the differences between the five universities and focusing on the benefits offered to dual-career students. Once all the benefits offered by the five universities had been identified, an initial list of benefits was sent to all members of the consortium for review. The universities provided feedback and expressed the need to better understand the benefits offered by the other universities in the consortium. Therefore, based on the feedback provided, the final list of 31 benefits was created and all 31 benefits were defined (Supplementary Material S2). This list, together with the definitions, was sent as a pilot-test and finally approved by the whole consortium.

The information obtained in the qualitative interviews and from the dual career questionnaire was used to create a list of the benefits for dual-career student athletes at the consortium's five universities. The list of benefits was sent to the project partners for validation. The interpretation and definition of each of the benefits was adjusted and a final list of 31 benefits was created (Supplementary Material S2). The consortium has grouped the 31 benefits into four categories according to their content:

1. All those benefits that directly affect their academic studies: Academic benefits (15 benefits).
2. All those benefits that focus on sporting activity and facilitate student athletes training or studies: Sports benefits (5 benefits).
3. All the benefits that have a health or fitness aspect: Health-related benefits (6 benefits).
4. All the benefits of different categories that favour the development of the students' dual career and that do not clearly fall into the three previous categories: Other benefits (5 benefits).

In the second phase of the study, which took place from January 10 to 20, 2023, a questionnaire was developed for the staff of the sports services and international relations offices to evaluate the list of 31 benefits. The questionnaire consisted of three main questions. Since the respondents were experts, they were asked to rate two of the questions according to three levels of relevance. The questionnaire asked participants to rate:

1. The importance of each benefit, regardless of whether their university offered that benefit or not. Scoring ranged from 1 to 3: 1 = not very important; 2 = important; 3 = very important.
2. Ease of implementing that benefit. Scoring ranged from 1 to 3: 1 = difficult to implement; 2 = fairly easy to implement; 3 = very easy to implement.
3. Whether the university offered the benefit. In this case, the possible responses were: yes, no or DK/NA.

The third phase of the study, from January 23, 2023 to February 28, 2023, focused on the dual-career students attending the universities participating in the SAMEurope project. In this phase, a detailed survey was developed using the Qualtrics® tool and it was approved and validated by the five universities in the consortium. The aim of the survey was to gather information about the dual-career students at the five universities, with a special focus on the needs of this group. Each partner university contacted its dual-career students and sent them the link to the Qualtrics® survey. The survey consisted of 27 questions divided into six sections:

1. Consent (1 question): agreement to participate in the study.
2. Personal information (3 questions): descriptive data about the dual-career student athlete.
3. Academic information (6 questions): data on their level of study and field of knowledge.
4. Athletic information (9 questions): data on the sport they play and their level of play.
5. Dual career (5 questions): data on the combination of academic and sporting life of dual-career student athletes, in particular the importance of the benefits received as dual-career students and their use of these benefits.
6. Erasmus (3 questions): data on the relevance of international mobility for dual career students.

The survey was written in English and translated into the national languages of the five consortium countries: German, Spanish, Finnish, French and Swedish. Each participant could choose to complete the survey in one of these six languages.

This paper focuses on the dual career section, in particular on the two questions about the benefits that universities provide to their dual-career students (Question 1: Rate from 0 to 5 the importance of each of these benefits in order to be able to combine your university studies and your high-level sport (0 = not at all important, 5 = very important); Question 2: Indicate which benefit or benefits you have used). The aim was to assess the benefits' importance and use. Dual-career students were asked to rate, from 0 to 5, each of the 31 benefits identified by the SAMEurope project (0 being not important and 5 being very important). They were then asked to indicate whether or not they had used each of the benefits.

### 2.3 Data analysis

**Figure 1:**
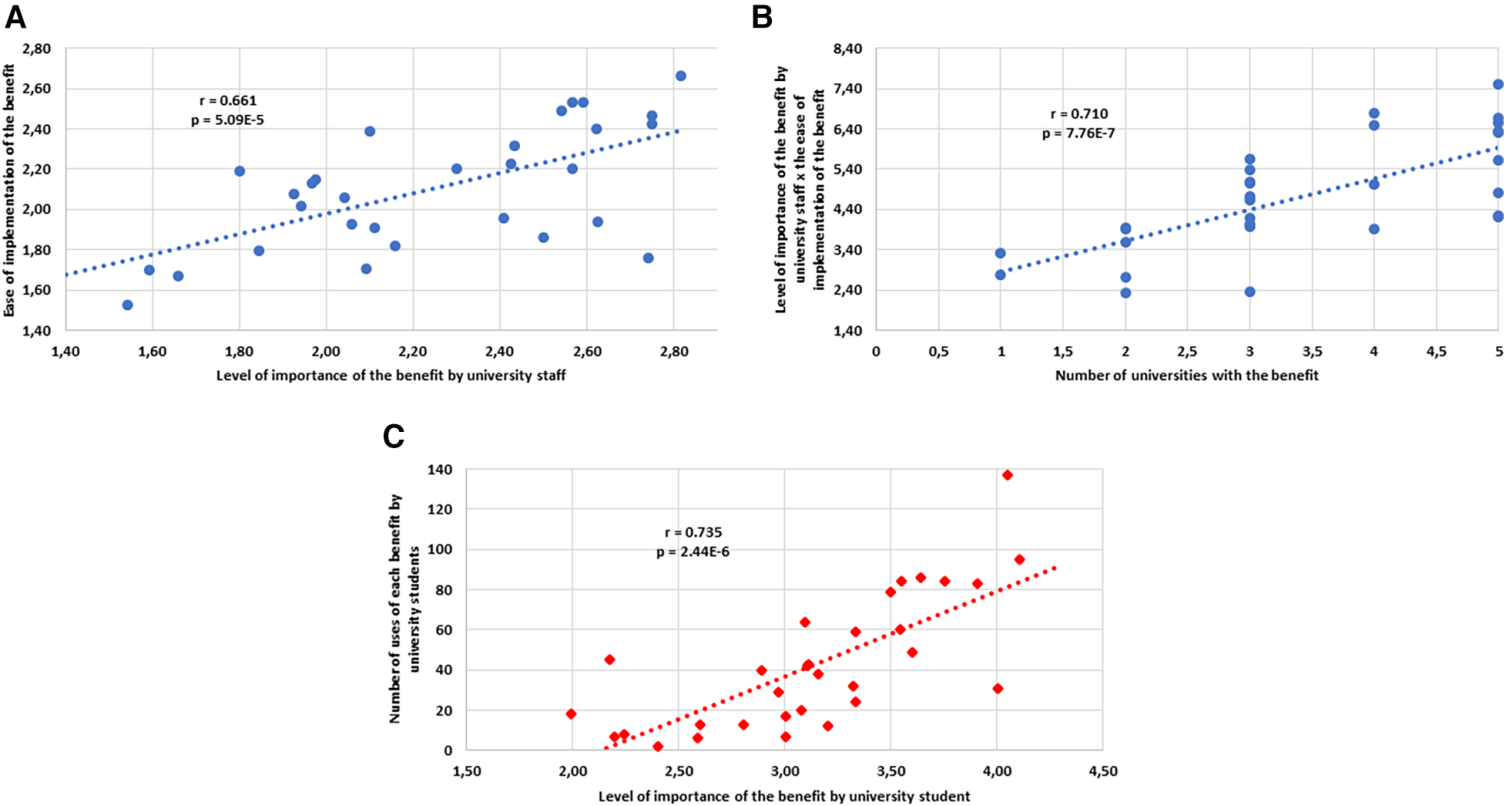
Correlations. (**A**) Correlation between the level of importance of the benefit by university staff and the ease of implementation of the benefit. (**B**) Correlation between the level of importance of the benefit by university staff x the ease of implementation of the benefit and the number of universities with the benefit. (**C**) Correlation between the level of importance of the benefit by university students and the number of uses of each benefit by university students. *r*, Pearson correlation; p, *p* value.

**Figure 2:**
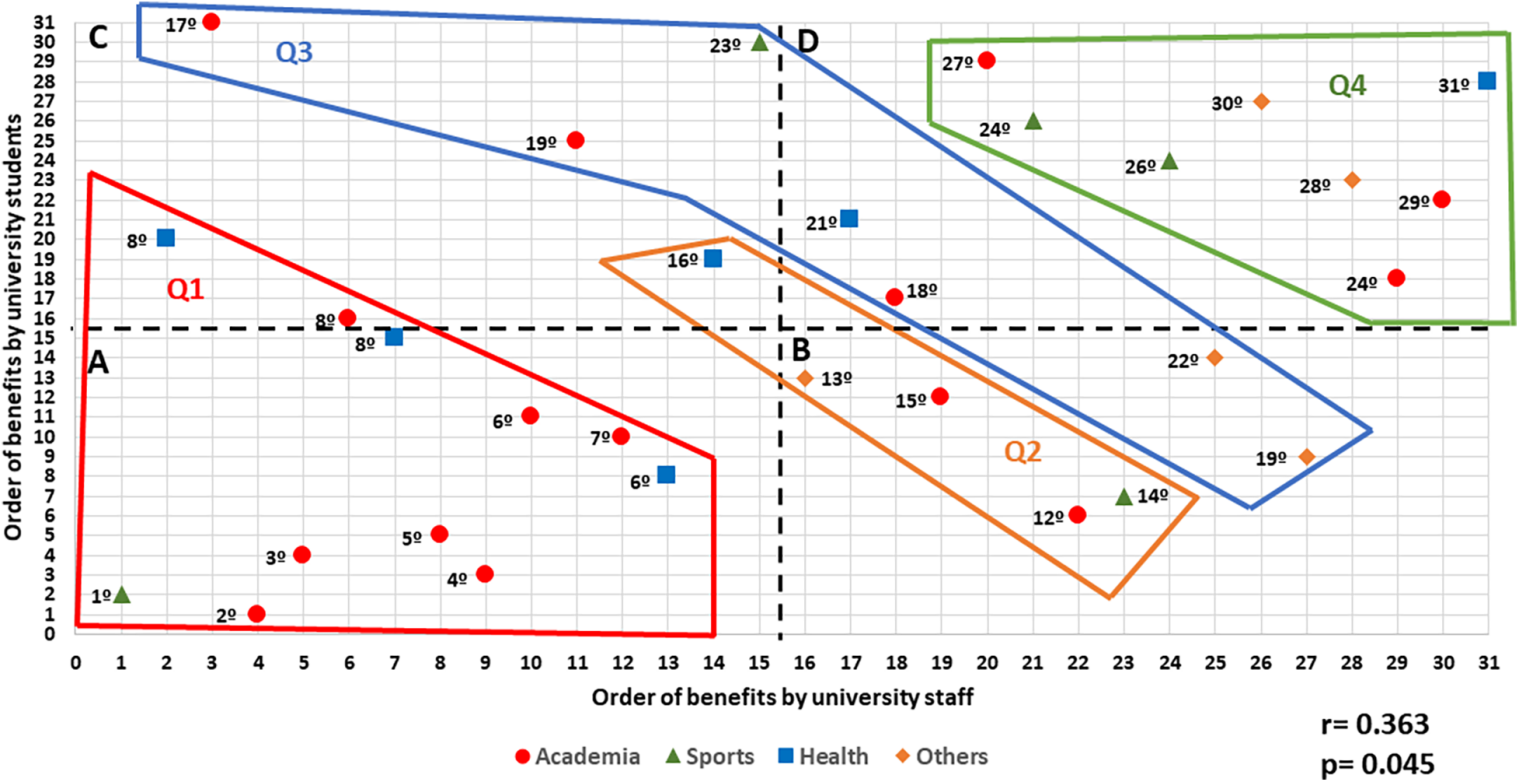
Correlation between the order of benefits by university staff and the order of benefits by university students. (**A**) Panel with the most important benefits for students and staff (**B**) Panel with the most important benefits for students and less important benefits for staff. (**C**) Panel with the least important benefits for students and the most important benefits for staff. (**D**) Panel with the least important benefits for students and for staff. Q1 Quartil 1, Q2 Quartil 2, Q3 Quartil 3 y Q4 Quartil 4. The numbers indicate the position of each benefit in the overall rankings, taking into account both the student and the staff perspectives (Table 5). *r*, Pearson correlation, p, *p* value.

Statistical analyses were performed using the SPSS v27 software, and two-sided values of *p* < 0.05 were considered statistically significant. The Kolgomorov-Smirnov test was used to test the normality of the data for all variables used. Mean and standard deviation were used to describe the collected data in terms of continuous variables, e.g., the value of a benefit or ease of implementation and sample size for categorical variables, e.g., number of times a benefit was used or number of universities offering a benefit. In the case of quartiles, Tukey's Hinges were used. A Pearson correlation test was used to analyze the intra- and inter-group correlations (Figures 1, 2). Cohen's criterion was used to determine the degree of correlation, whereby correlations with a value between 0.1 and 0.29 are considered weak correlations, correlations between 0.30 and 0.50 are moderate correlations, and correlations above 0.50 are strong correlations (28).

## 3 Results

Our study was able to identify and define 31 different benefits offered by the universities participating in the SAMEurope project (Table 1). These benefits have been grouped into four blocks: (i) Block 1 focuses on academic benefits that help students pursue their studies alongside their sports activity (15 benefits); (ii) Block 2 (5 benefits) aims at helping the student athletes pursue their sports training; (iii) Block 3 (6 benefits) aims at helping the student athletes maintain their condition and health throughout their dual career; and (iv) Block 4, referred to as “others” (5 benefits) are aspects that help to make the student athlete's daily life easier.

**Table 1:** Set of 31 benefits defined in the five universities of the SAMEurope project to facilitate the implementation of the dual career for high-level student athletes.

Academic benefits	Choose class groups
	Justification for absences
	Online courses
	Remedial courses
	Specific courses (such as time management, sports marketing or social networks)
	Changing exam dates
	Online exams (without changing dates)
	Adaptation of the pace of study
	Extesion of the number of exam sessions
	Extension of the criteria for permanency
	Partial enrollment
	Free semesters
	Separate academic group
	Academic tutoring
	Career advice
Sports benefits	Free use of sports facilities
	Private use of sports facilities
	Reservation of places for sports courses
	Extra credits for participation in university sport events
	DC tutoring
Health-related benefits	General medical services
	Mental health support
	Physiotherapy
	Nutricionist
	Testing (physiology, biomechanics, performance)
	Specialized PE teachers
Other benefits	Housing
	Discounts on meals
	Adapted catering service
	Scholarships
	Extra points in Erasmus evaluation

The set of 31 benefits illustrates the way in which the universities of the SAMEurope project support and assists these dual-career athletes at their universities. A description of each benefit is provided in Supplementary Material S2.

The 31 benefits described above were defined in three phases. The first phase involved 10 experts and the second phase involved 24 experts from the five universities in the consortium (three from Chalmers University of Technology, eight from the Institut National des Sciences Appliquées de Lyon, three from the Karlsruhe Institute of Technology, two from the University of Jyväskylä and eight from the Universitat Jaume I de Castellón).

The third phase involved 208 dual-career students from the five universities, 40.47% of the total number of students enrolled in the dual-career programs at these universities. Table 2 shows the distribution of the dual-career athletes from the five universities and the participation of the students.

**Table 2:** Distribution of dual career students who participated in the survey conducted by the five universities of the SAMEurope project.

University	Chalmers	INSA	KIT	JYU	UJI	General
Questionnaires sent	103	150	26	140	95	514
Questionnaires received	32	89	15	22	50	208
Age	22.16 ± 1.34	21.54 ± 0.70	22.67 ± 2.81	23.09 ± 3.07	21.16 ± 5.76	21.79 ± 1.77
Men	13	31	11	17	19	91
Women	19	58	4	5	30	116
Non binary					1	1
Bachelor degree	32	42	11	16	49	140
Master degree		47	4	6	1	68
Regional level		1		1	1	3
National level	14	47		15	41	119
International level	18	41	15	6	8	86
% participation	31.07%	59.33%	57.69%	15.71%	52.63%	40.47%

Chalmers, Chalmers University of Technology; INSA, Institut National des Sciences Appliquées de Lyon; KIT, Karlsruhe Institute of Technology; JYU, University of Jyväskylä; UJI, Universitat Jaume I.

The responses provided by the 24 dual-career experts from the different universities made it possible to rank the 31 benefits in terms of significance, taking into account three variables: the benefit's importance, its ease of implementation, and the number of universities that offer the benefit to their students. This ranking, divided into quartiles (Table 3), allows us to determine which benefits are the most relevant for the university staff participating in the survey. Thus, the three most important benefits from the universities' point of view were the free use of sports facilities, general medical services, and career advice. The three least important benefits were being part of a separate academic group, offers for remedial courses, and access to specialized physical education teachers. Note that the last two are benefits that are only available at one of the five universities participating in the SAMEurope project.

**Table 3:** Ranking of the benefits from the perspective of the university staff, taking into account the importance of the benefit, the ease of its implementation, and the number of universities offering it.

BLOCK	Benefit	LI	EI	NUni	LI x EI	LI x EI x NUni	OB	Q
SPORTS	Free use of sports facilities	2.82 ± 0.43	2.67 ± 0.44	5	7.51	37.56	1	1
HEALTH	General medical services	2.75 ± 0.39	2.43 ± 0.39	5	6.67	33.34	2	1
ACADEMIA	Career advice	2.59 ± 0.24	2.53 ± 0.29	5	6.57	32.83	3	1
ACADEMIA	Justification for absences	2.54 ± 0.41	2.49 ± 0.39	5	6.33	31.66	4	1
ACADEMIA	Adaptation of the pace of study	2.62 ± 0.66	2.40 ± 0.83	5	6.30	31.50	5	1
ACADEMIA	Academic tutoring	2.43 ± 0.41	2.32 ± 0.55	5	5.64	28.19	6	1
HEALTH	Mental health support	2.75 ± 0.41	2.47 ± 0.62	4	6.78	27.13	7	1
ACADEMIA	Choose class groups	2.57 ± 0.43	2.53 ± 0.41	4	6.50	26.01	8	1
ACADEMIA	Changing exam dates	2.74 ± 0.74	1.76 ± 0.70	5	4.82	24.10	9	2
ACADEMIA	Extension of number of exam session	1.98 ± 0.49	2.15 ± 0.45	5	4.25	21.23	10	2
ACADEMIA	Specific courses (e.g., time management, sports marketing & social media)	2.04 ± 0.15	2.06 ± 0.17	5	4.20	21.01	11	2
ACADEMIA	Partial enrollment	2.10 ± 0.28	2.39 ± 0.68	4	5.02	20.09	12	2
HEALTH	Physiotherapy	2.57 ± 0.42	2.20 ± 0,54	3	5.65	16.94	13	2
HEALTH	Nutritionist	2.43 ± 0.47	2.23 ± 0.42	3	5.40	16.19	14	2
SPORTS	Reservation of places for sports courses	1.94 ± 0.54	2.02 ± 0.58	4	3.92	15.66	15	2
OTHERS	Housing	2.63 ± 0.49	1.94 ± 0.39	3	5.10	15.29	16	3
HEALTH	Testing (physiology. biomechanics. performance)	2.30 ± 0.36	2.20 ± 0.34	3	5.06	15.18	17	3
ACADEMIA	Online courses	2.41 ± 0.53	1.96 ± 0.37	3	4.72	14.15	18	3
ACADEMIA	Online exams (without changing dates)	2.50 ± 0.55	1.86 ± 0.59	3	4.65	13.94	19	3
ACADEMIA	Free semesters	1.97 ± 0.42	2.13 ± 0.36	3	4.20	12.59	20	3
SPORTS	DC tutoring	2.11 ± 0.72	1.91 ± 0.66	3	4.03	12.09	21	3
ACADEMIA	Extension of the criteria for permanency	1.93 ± 0.42	2.08 ± 0.43	3	3.99	11.98	22	3
SPORTS	Private use of sports facilities	2.06 ± 0.06	1.93 ± 0.27	3	3.96	11.89	23	3
SPORTS	Extra credit for participation in university sport events	1.80 ± 0.66	2.19 ± 0.81	2	3.95	7.89	24	4
OTHERS	Scholarships	2.16 ± 0.57	1.82 ± 0.55	2	3.92	7.84	25	4
OTHERS	Extra points in Erasmus evaluation	2.09 ± 0.29	1.71 ± 0.33	2	3.57	7.15	26	4
OTHERS	Adapted catering service	1.54 ± 0.28	1.53 ± 0.27	3	2.35	7.05	27	4
OTHERS	Discounts on meals	1.59 ± 0.41	1.70 ± 0.80	2	2.71	5.41	28	4
ACADEMIA	Separate academic group	1.36 ± 0.12	1.72 ± 0.26	2	2.33	4.66	29	4
ACADEMIA	Remedial courses	1.84 ± 0.53	1.80 ± 0.73	1	3.31	3.31	30	4
HEALTH	Specialized PE teachers	1.66 ± 0.62	1.67 ± 0.53	1	2.76	2.76	31	4

Values for importance of benefit and ease of implementation are presented as means and standard deviation. LI, level of importance of the benefit by university staff; EI, ease of implementation of the benefit; NUni, number of universities with the benefit; LI x EI, level of importance of the benefit x ease of implementation of the benefit; LI x EI x NUni, level of importance of the benefit x ease of implementation of the benefit x number of universities with the benefit; OB, order of benefits by university staff; Q, quartil.

With respect to the relationship of the results obtained from the participating university staff, we observed that there was a positive, strong and significant correlation (*r* = 0.661, *p* = 5.09E-5) between the benefit this staff considered to be important and the ease of implementing this benefit (Figure 1A). At the same time, there was a positive, strong and significant correlation (*r *= 0.710 *p* = 7.76E-7) between the product of the importance of the benefit and the ease of implementation and the number of universities that offered this benefit (Figure 1B).

Similarly, the group of 208 students who rated each of the benefits and who indicated whether or not they used the different benefits (183 students used some benefit, with an average of 6.37 ± 4.85 benefits used per student), provide us with a ranking of the different benefits from the perspective of the students (Table 4). The ranking of these benefits in quartiles shows that justification for absences, the free use of sports facilities, and the changing of exam dates were the three most relevant benefits for students, while free semesters, reservation of places for sports courses, and career advice were the three benefits of least interest to the students.

**Table 4:** Ranking of the benefits from the perspective of the dual-career university students, taking into account the importance of the benefit and the number of uses made of each benefit.

BLOCK	Benefit	LI	NUB	LI x NUB	OB	Q
ACADEMIA	Justification for absences	4.05 ± 1.36	137	555.25	1	1
SPORTS	Free use of sports facilities	4.11 ± 1.35	95	390.50	2	1
ACADEMIA	Changing exam dates	3.91 ± 1.40	83	324.42	3	1
ACADEMIA	Adaptation of the pace of study	3.75 ± 1.47	84	315.40	4	1
ACADEMIA	Choose class groups	3.64 ± 1.38	86	312.99	5	1
ACADEMIA	Extension of the criteria for permanency	3.55 ± 1.75	84	298.44	6	1
SPORTS	Private use of sports facilities	3.50 ± 1.54	79	276.50	7	1
HEALTH	Physiotherapy	3.54 ± 1.56	60	212.60	8	1
OTHERS	Adapted catering service	3.10 ± 1.73	64	198.15	9	2
ACADEMIA	Partial enrollment	3.33 ± 1.68	59	196.57	10	2
ACADEMIA	Extension of number of exam session	3.60 ± 1.62	49	176.45	11	2
ACADEMIA	Online exams (without changing dates)	3.12 ± 1.65	43	133.96	12	2
OTHERS	Housing	3.11 ± 1.68	42	130.44	13	2
OTHERS	Scholarships	4.00 ± 1.39	31	124.15	14	2
HEALTH	Mental health support	3.16 ± 1.54	38	120.03	15	2
ACADEMIA	Academic tutoring	2.89 ± 1.64	40	115.58	16	2
ACADEMIA	Online courses	3.32 ± 1.59	32	106.31	17	3
ACADEMIA	Separate academic group	2.17 ± 1.63	45	97.79	18	3
HEALTH	Nutritionist	2.97 ± 1.55	29	86.16	19	3
HEALTH	General medical services	3.33 ± 1.59	24	79.96	20	3
HEALTH	Testing (physiology. biomechanics. performance)	3.08 ± 1.62	20	61.54	21	3
ACADEMIA	Remedial courses	3.00 ± 1.60	17	51.08	22	3
OTHERS	Discounts on meals	3.20 ± 1.79	12	38.42	23	3
SPORTS	Extra credit for participation in university sport events	2.81 ± 1.82	13	36.50	24	4
ACADEMIA	Specific courses (e.g., time management, sports marketing & social media)	1.99 ± 1.51	18	35.83	25	4
SPORTS	DC tutoring	2.60 ± 1.67	13	33.81	26	4
OTHERS	Extra points in Erasmus evaluation	3.00 ± 1.73	7	21.03	27	4
HEALTH	Specialized PE teachers	2.24 ± 1.60	8	17.92	28	4
ACADEMIA	Free semesters	2.59 ± 1.80	6	15.55	29	4
SPORTS	Reservation of places for sports courses	2.20 ± 1.73	7	15.38	30	4
ACADEMIA	Career advice	2.40 ± 1.54	2	4.81	31	4

Values for benefit importance are presented as means and standard deviation. LI, level of importance of the benefit by university students; NUB, number of uses of each benefit by university students; LI x NUB, level of importance of the benefit by students x Number of uses of each benefit by students; OB, order of benefits by university students; Q, quartil.

In terms of intra-student correlations, the relationship between the value placed on the benefit by the students and the number of times they use it was positive, strong and significant (*r* = 0.735 *p* = 2.44E-6) (Figure 1C).

Once the benefits were ranked by the students and the staff at the universities, it was possible to obtain a general ranking of all 31 benefits. Thus, we obtained a global and joint view of the ranking of the benefits from the perspective of the staff and students, as well as results for the five universities that make up the SAMEurope project as a whole. These results, divided into quartiles (Table 5), show that the free use of sports facilities, justification for absences, and the adaptation of the pace of studies were the three most important benefits in the overall ranking of benefits. At the same time, the offer of remedial courses, extra points for Erasmus+ mobility, and specialized physical education teachers were the three least relevant benefits in the overall ranking.

**Table 5:** Ranking of the benefits from the perspective of the university staff and the dual-career students of the five universities, taking into account the final ranking of each of the perspectives.

BLOCK	Benefit	OBSta	OBStu	GR	GO	Q
SPORTS	Free use of sports facilities	1	2	3	1	1
ACADEMIA	Justification for absences	4	1	5	2	1
ACADEMIA	Adaptation of the pace of study	5	4	9	3	1
ACADEMIA	Changing exam dates	9	3	12	4	1
ACADEMIA	Choose class groups	8	5	13	5	1
ACADEMIA	Extension of number of exam session	10	11	21	6	1
HEALTH	Physiotherapy	13	8	21	6	1
ACADEMIA	Partial enrollment	12	10	22	8	1
HEALTH	General medical services	2	20	22	8	1
ACADEMIA	Academic tutoring	6	16	22	8	1
HEALTH	Mental health support	7	15	22	8	1
ACADEMIA	Extension of the criteria for permanency	22	6	28	12	2
OTHERS	Housing	16	13	29	13	2
SPORTS	Private use of sports facilities	23	7	30	14	2
ACADEMIA	Online exams (without changing dates)	19	12	31	15	2
HEALTH	Nutritionist	14	19	33	16	2
ACADEMIA	Career advice	3	31	34	17	3
ACADEMIA	Online courses	18	17	35	18	3
ACADEMIA	Specific courses (e.g., time management, sports marketing & social media)	11	25	36	19	3
OTHERS	Adapted catering service	27	9	36	19	3
HEALTH	Testing (physiology, biomechanics, performance)	17	21	38	21	3
OTHERS	Scholarships	25	14	39	22	3
SPORTS	Reservation of places for sports courses	15	30	45	23	3
ACADEMIA	Separate academic group	29	18	47	24	4
SPORTS	DC tutoring	21	26	47	24	4
SPORTS	Extra credit for participation in university sport events	24	24	48	26	4
ACADEMIA	Free semesters	20	29	49	27	4
OTHERS	Discounts on meals	28	23	51	28	4
ACADEMIA	Remedial courses	30	22	52	29	4
OTHERS	Extra points in Erasmus evaluation	26	27	53	30	4
HEALTH	Specialized PE teachers	31	28	59	31	4

OBSta, order of benefits by university staff; OBStu, order of benefits by university students; GR, general results by staff and students; GO, general order benefits by staff and students; Q, quartil.

Finally, when analyzing the correlation between the ranking of the benefits by the staff and by the students, we found that there was a positive, moderate and significant relationship (*r* = 0.363 *p* = 0.045) (Figure 2).

## 4 Discussion

The main objective of this study was twofold: to identify and to define the benefits offered to dual-career students at the five universities participating in the SAMEurope project. Once the benefits were defined, they were ranked according to their relevance from the perspective of the staff and the dual-career students. Then the level of the relationship within groups (staff and students) and between groups (staff vs. students) was determined.

The hypothesis is not fulfilled, as there is a correlation between the students' and the staff's ranking of benefits. However, this correlation has a very low value (*r* = 0.363, *p* = 0.045), in contrast to the *p*-values found within each of the groups (*r* between 0.661 and 0.735, *p* < 0.001), indicating that the relationship between the staff's ranking of importance and the dual-career students' ranking of those benefits has the potential to be improved by direct action by higher education institutions.

In the first part of the study, 31 benefits offered to dual-career students at the different universities in the consortium were identified and defined (Table 1 and Supplementary Material S2). These 31 benefits are in line with those presented by Izzicupo et al. (29), who defined 26 different aspects. In this study, the 31 benefits were analyzed, described and defined by experts working directly in the dual-career field at each of the five universities. In contrast to the study by Izzicupo et al. (29), the benefits were implemented and applied at the universities in the consortium. Each of these 31 benefits (Table 1) directly served the dual-career students pursuing their academic education at one of these universities. As can be seen in the results of the staff (Table 3), not all of the 31 benefits were implemented at all universities. Some benefits are offered by all five universities, such as justification for absences or academic tutoring, and others are offered by only one university, such as specialized physical education teachers and remedial courses. These differences in the availability of the benefits are based on the context of each university, the social culture in which the university is located, legislation, and national models for support for elite athletes. The legislative framework of each country has a direct influence on policies to support elite athletes. However, university autonomy, which is a characteristic element of universities, allow for modifications and adaptations within the university regulations. Thus, there are universities that have a more open program towards the protection of dual-career athletes and other universities that are more restricted to the development of dual-career programs. There is a need to create a framework for action in the European Union aimed at the continuity of higher education studies and high-performance sport. Aquilina and Henry (14), and other subsequent authors (6, 15, 30) already point to differences in national models of support for dual-career students, which range from models with more state involvement, to models without a defined structure to support this group.

After the 31 benefits were defined and grouped into 4 areas, the benefits were ranked by asking for the opinion of the university staff with regard to each of the benefits (Table 3). Since these were experts, a very well-defined scale was used, with little room for indecision, to allow them to clearly position themselves with regard to the importance of the benefit and the ease of its implementation at their university. The experts were asked to provide their opinion to every question, regardless of whether or not their university offered the benefit.

Using this data, the results for each question were combined to produce an overall ranking of the benefits from most important to least important based on the responses from the participating university staff (Table 3). This model combines three questions asked in such a way that the ranking of the benefits takes into account how important the benefit is, how easy it is to implement at each university (the less easy it is, the more complex it is to implement) and the number of universities that offer the benefit (the more universities offering the benefit, the more relevant it is, because it is more widely implemented within the university system of the different universities). Thus, in contrast to the study by Izzicupo et al. (29), which ranks the 26 aspects taking into account the relationship between the importance of the benefit and the ease of implementation, and thus obtaining 4 quadrants for the location of the 26 aspects, our model manages to obtain a global ranking, taking into account three variables. However, both models have found that the most valued benefits are centered around aspects relating to logistics, academics and tutoring. In our case, the relevance of medical support for dual-career students was also included in the assessment for the first time. The results are presented in quartiles (Table 3) so that we can observe the ranking of the benefits from the perspective of the staff in four quartiles. The first quartile shows the importance of mental health support. The second quartile shows the importance of physiotherapy and the nutritionist. These benefits, considered important by university staff, have not previously been taken into account when assessing the support received by dual career athletes (8, 10, 31).

The results were correlated in order to see the relationship between the importance of the benefit and its ease of implementation. We found that there is a positive, strong and significant relationship between both variables (Figure 1A). This leads us to believe that the expert personnel try to facilitate the implementation of those benefits which they consider to be important and to define action policies adapted to their own context. In this way, they implement tailor-made measures and policies that benefit dual-career students.

We also analyzed the correlation between the importance and ease of implementation with the number of universities offering this benefit. We found that there is a positive, strong and significant relationship between both variables (Figure 1B). Thus, higher scores for the importance of the benefit and for the benefits that are very easy to implement result in a greater number of universities offering these benefits. Thus, in contrast to the work of Izzicupo et al. (29), we find that the greater or lesser number of universities offering a particular benefit is related to the degree of importance of the benefit and its ease of implementation in each university. This combination of factors is an element that helps to understand why some benefits are more widely offered in more universities than others. Knowing the tools for implementing a benefit at one university paves the way for others to make it easier to implement it.

Similarly, the benefits were ranked from the student's perspective. It is important to note that the questionnaire was available in six languages so that each student could choose to complete the questionnaire in his or her own language. This facilitated the understanding of each benefit. When ranking the benefits from the students' perspective, responses to two questions were analyzed, one relating to the importance of the benefit and the other focusing on which benefits they had used. Thus, both variables were taken into account in the final ranking of the benefits. In recent studies, student surveys have been carried out qualitatively in focus groups (17) and quantitatively (19–21), in which the needs and interests of the students in being able to pursue an optimal dual career are shown in different sections. Our study focused on understanding their perceptions of the benefits offered to them by their universities. The dual-career students rated each of the benefits according to their relevance and indicated which benefits they had used in developing their dual career. For these two results, each benefit was ranked according to its relevance as a whole at the universities participating in the SAMEurope project. Among the 6 most relevant benefits for students, 5 of them are in the ACADEMIA section (they are benefits related to students' time management) and 1 benefit is in the SPORTS section (the free use of sports facilities, which also directly affects students' time management). This fact is addressed in (32, 33), and it is in line with our results, pointing out that the most relevant aspect for students is to be able to manage their time. It can be seen that justification for absences stands out in this group, an aspect that has been shown to be important in the studies mentioned above (17, 19–21, 32, 33). Our study confirms its importance and its use by the dual-career students at the five universities (Table 4). It is also worth highlighting the scholarship benefit, which is ranked highly by the students but not utilized much, a fact that is conditioned by the number of universities that offer this benefit. Thus its final position in the ranking is much lower than the importance it has for this group. This shows the diversity of university policies and reinforces the autonomy of universities to make decisions tailored to the interests and needs of dual-career students. However, academic benefits continue to carry significant weight, albeit with a more practical and immediate impact on the students' daily lives, as with sports and health-related benefits.

In the case of the students, the relationship between the importance of the benefit and its use is positive, strong and significant (Figure 1C). Thus we see that the students are very consistent and utilize what they consider to be important, whenever it is offered. Therefore, the more the dual-career program is tailored to their interests, the more these benefits can be expected be utilized. The scientific literature has not addressed the question of how many benefits a university dual-career athlete uses. Our study is the first to address this issue. Each student athlete used 6.37 ± 4.85 different benefits, with a range of up to 31 benefits to choose from. The fact that not all benefits were offered by all universities may have influenced the number of benefits used.

After the analysis process, the 31 benefits were ranked, taking into account the three variables analyzed from the perspective of the staff and the two variables analyzed from the perspective of the students. An overall ranking was obtained for the group of experts and students from the five universities that make up the SAMEurope consortium (Table 5). The overall ranking shows eleven benefits in Quartile 1 (seven from the academic block, one from the sports block and three from the health block), five benefits in Quartile 2 (two from the academic block, one from the sports block, one from the health block, and one from the “other” block), seven benefits in Quartile 3 (three from the academic block, one from the sports block, one from the health block and 2 from the “other” block) and eight benefits in Quartile 4 (three from the academic block, two from the sports block, one from the health block, and two from the “other” block). The results show, for the first time, a system that ranks the set of benefits offered by different universities and from different countries globally and jointly, taking into account the two main actors involved in the development of dual careers for students at higher education institutions. This ranking can help to define the minimum benefits needed to be offered by an international dual-career program. Condello et al. (34) have already proposed this measure as a way to ensure the development of dual careers. Thus, the results obtained can act as a guide for defining the minimum benefits to be implemented at European universities.

Finally, a correlation was made between the ranking of the 31 benefits from the perspective of the staff and the students in order to verify the relationship between the two groups. This is the first time that such a correlation has been made. Previous studies have focused on both groups, staff and students, separately (19–21, 29, 31, 35). In this study, both groups are catered for at the same time. The result showed a positive, moderate and significant correlation (Figure 2), which leads us to assume that there is room for improvement in developing dual careers at higher education institutions and that the relationship between the two groups can be improved. Taking into account these results, improvements in the implementation of those benefits that are most relevant to the interests of the student athletes will enhance the relationship between the university and the dual-career student athletes, which, in turn, will promote the development of dual-career programs at European universities. Thus, a program more tailored to their interests and needs would increase the number of different benefits used by dual-career student to facilitate the compatibility of their academic life with their sporting life. The role of university autonomy is key to the promotion and development of these policies, as well as their influence on higher level regulations, such as national or EU policies.

Figure 2 shows the distribution of benefits in four panels. Panel A shows the benefits that are important to students and staff at the five universities. Panel B shows the benefits that are most important to students and least important to the staff. Panel C shows the benefits that are important to the staff but less important to the students. And, finally, Panel D shows the benefits ranked least important by the staff and students.

When analyzing the results obtained in the global ranking, it is observed that in Quartile 1 there are seven academic benefits, one of which is located in Panel C, three health-related benefits, one of which is located in Panel C, and one sports benefit, which tops the global ranking. In Quartile 2, there are two academic benefits, one sports benefit and one benefit from the “other” section located in Panel B, and one health-related benefit located in Panel C. In Quartile 3, the most dispersed, there are three academic benefits—two of which are located in Panel C and one of which is in Panel D, one sports benefit located in Panel C, and two benefits from the “other” section that are in Panel B. Finally, Quartile 4 has three academic benefits, two sports benefits, two health-related benefits and one benefit from the “other” category, all located in Panel D.

Figure 2 helps to show which benefits need more decisive measures to bring the interests of the students more in line with those of the university. For example, the career advice benefit, located in Panel C and in Quartile 3 and ranked 17, requires measures to make students see this benefit as something useful for their professional future. On the other hand, the scholarship benefit, located in Panel B and in Quartile 3 and ranked 22, seems to be an element that can improve the rapprochement between both groups involved in the development of dual careers. Our analysis reinforces the need to combine the interests of both groups and to take a step forward with respect to publications that separately address the interests of the different collectives (17, 19, 29, 34).

One of the elements to consider is the search for consensus in defining what an elite athlete is. This highlights the fact that there are differences in the interpretation of what an elite athlete is. Some studies make efforts to define this (36, 37) and can provide a tool for establishing a consensus in the definition of elite athlete between different higher education institutions and in different countries or contexts. This, in turn, would help to facilitate the recognition and mobility of student athletes between universities.

In this study we have listed and ranked a total of 31 benefits aimed at facilitating the combination of higher education and elite sport. These 31 benefits were ranked from the perspective of the two groups directly involved in the dual career at universities: the student athletes and the university staff working with dual-career athletes. This ranking of the 31 benefits can serve as a guide for higher education institutions to select those benefits that are more decisive in the pursuit of a dual career and to opt for programs that are more in line with the interests and needs of both groups.

### 4.1 Limitations

One of the limitations of this study was the number of dual-career students who participated in the survey. Even though 52.14% participated, only 40.46% completed the questionnaire. Higher participation would strengthen the results and it would be advisable to increase the number of participating students. Similarly, the participation of only five universities in the study is another limitation and it would be advisable to reproduce the study with a larger number of European universities.

Another limitation is the possible misinterpretation of the meaning of the different benefits by the students, however an attempt was made to reduce potential misinterpretations by conducting the survey in six languages, one in English and one for each country in the consortium.

This study shows that the structural profiles of each country lead to different academic policies. Therefore, within the framework of the European Union, studies focusing on the individual reality of each institution/country will facilitate the creation of programs to promote the mobility of all high-level student athletes in higher education institutions and to support their international mobility within the framework of the European Union.

One limitation to bear in mind is that this study has focused on the two most representative groups of dual career in higher education institutions, students and university staff (faculty and technical staff), but in the field of high-level sport, it may be necessary to include in this analysis the group of coaches who are responsible for the training programs of dual-career student athletes.

### 4.2 Conclusions

This paper identifies 31 benefits offered to student athletes at one or more of the universities in the SAMEurope consortium to facilitate the development of their dual career. A ranking of the benefits was established from the perspective of the participating staff members and from the perspective of the students. In addition, *a priori*tized ranking of the 31 benefits was established by combining the two perspectives across the five universities in the consortium. A strong positive relationship was observed between the importance of the benefits and the ease of implementation by the university staff. Furthermore, this positive and strong relationship is also present with regard to the number of universities offering the benefits. Similarly, there is a strong positive relationship between the importance of the benefits and their use by the students. However, there is only a moderate relationship between the ranking of the benefits among the staff and the students. Thus, there is significant room for improvement to better align the benefits offered by the universities to the priorities of the students.

The marked differences in the definition of elite athlete in each country make it difficult to agree on a single criterion for the university community. The establishment of a consensus definition will make it easier for higher education institutions to find a stable criterion for standardization.

## Data Availability

The datasets presented in this article are not readily available because the traceability of responses can lead to the identification of the subjects. Partial data are available upon request to the corresponding author. Requests to access the datasets should be directed to CH, hernando@uji.es.
