# Supplementary material for: Promoting dual careers at higher education institutions: 31 benefits ranked by the project Student Athletes Erasmus+ Mobility in Europe (SAMEurope)
Source: Front Sports Act Living. 2024 Jul 1;6:1407194. doi: 10.3389/fspor.2024.1407194 (PMC11246954; doi:10.3389/fspor.2024.1407194)
Supplement: Supplementary file 1 [file Datasheet1.pdf]

Supplementary Material 1

Semi-structured qualitative interviews with sport technician staff who care for dual career athletes at each of the universities in the consortium

1. Description of the elite athlete programme

a. Year of creation of the programme

b. Responsible person (name, title, e-mail address)

c. Persons working full or part-time on the programme

d. Number of students taking part in the programme per year (if there is a limit)

e. Number of students who have joined the programme since its creation.

f. Number of student athletes who have benefited from the dual career programme in the 2021-2022 academic year.

g. Is the programme endorsed by the university's governing body? How?

h. Does the university receive state, regional or private financial support for the management of the programme?

i. Financial budget allocated to the programme.

2. Access to the programme

a. What are the requirements for a student athlete to enter a dual career programme or, alternatively, to obtain benefits to combine his or her studies with his or her training/competition programme?

b. Is information about the dual career publicly available? Please indicate where this information can be found and provide the link to the website or, failing that, a copy of the information.

c. What documentation do you have to submit?

d. Where do you submit the documentation?

e. Is there a deadline for inclusion in the programme? If so, please specify.

f. Who reviews the documentation submitted?

g. Who informs the student-athlete of the outcome of his or her application?

h. What is the deadline for communicating the result?

i. Is the outcome of the application subject to appeal?

j. Does membership in the programme expire, how long does it last, can it be renewed, how many times can it be renewed?

k. Is any sport accepted, whether individual or team?

l. Are there any subjective criteria when including a student athlete in the dual career?

m. Who makes the decision to include a student athlete in the dual career if there are subjective criteria?

3. What are the academic benefits of the dual career? Explain the benefit, how it is obtained and how it is carried out. For instance:

a. Preference to choose the groups that best suit their training.

b. Possibility of changing dates of official exams if they coincide with a competition or an activity related to their status as an elite athlete.

c. Financial assistance in services (specify, for example, if they will have a reduction in the sports card, meals, courses organised by the sports service...).

d. Justification for absences due to attendance to competitions or activities related to their status as elite sportsperson.

e. Personal academic tutoring.

4. What are the sports benefits of belonging to the dual career programme? For instance:

a. Use of sports facilities (gymnasium, swimming pool, etc.).

b. Reservation of 10% of the places on training courses organised by the sports department.

5. Are there other benefits related to a dual career? For instance:

a. Granting of scholarships depending on the results obtained.

b. Medical benefits / Medical allowances

6. Relationship with student athletes

a. Student-athlete feedback: Is there any kind of survey to assess student-athlete satisfaction?

b. Do student athletes have to meet the requirements of the host university in order to benefit from the dual career programme or is it sufficient that they are dual career students at their home university?

c. Do the exchange student athletes have the same benefits as student athletes at the host university?
