# Supplementary material for: Promoting dual careers at higher education institutions: 31 benefits ranked by the project Student Athletes Erasmus+ Mobility in Europe (SAMEurope)
Source: Front Sports Act Living. 2024 Jul 1;6:1407194. doi: 10.3389/fspor.2024.1407194 (PMC11246954; doi:10.3389/fspor.2024.1407194)
Supplement: Supplementary file 2 [file Datasheet2.pdf]

Supplementary Material 2

Description of the 31 identified benefits

ACADEMIC BENEFITS

Choose class groups: Dual-career student athletes can have the flexibility to choose class groups that fit their training and competition schedules. This allows them to organize their academic load more effectively, adjusting it to their training and study needs.

Justification for absences: Since student athletes may need to be absent from classes due to sports commitments (competitions and training camps), they are provided with a formal process to justify these absences. This allows them to keep up with their studies and make up missed material in an appropriate manner.

Online courses: Online courses can be offered to allow student athletes to access academic content remotely, especially when they are traveling to compete or attend official training camps. These courses provide them with the flexibility to keep up with their studies from any location.

Remedial courses: In the event that student athletes are forced to miss classes due to official competitions or training camps, make-up courses are offered to help them catch up on the academic content taught during the period of absence. These additional courses give them the opportunity to maintain their academic progress without significant delays.

Specific courses: Specific courses designed to address the unique needs of student athletes are offered, such as time management, sports marketing or social networking. These courses provide them with additional skills that are beneficial both in their athletic career and in their personal and professional development.

Changing exam dates: To accommodate the sporting commitments of student athletes, the exam schedule is adjusted to their competition and official training camp schedules, modifying, if necessary, the dates of official exams. This gives them the opportunity to concentrate on their competitions and to be able to take their exams at a more suitable date.

Online exams (without changing dates): Students are given the opportunity to take exams online without rescheduling, allowing them to keep their academic calendar uninterrupted while participating in sporting events or official training camps. In this case, they take the exams from their place of concentration or competition, following a protocol that guarantees that the requirements of an official exam are maintained.

Adaptation of the pace of study: The planning of academic activities is adjusted so that student athletes can manage their sports and academic commitments in a balanced way. It also implies the possibility of registering for fewer credits than those recommended for general students.

Extension of the number of exam sessions: In cases where there is a limit on the number of exams calls to pass a subject, the number of official exams calls for final exams may be increased.

Extension of the criteria for permanency: Where there are minimum requirements to continue studying, the permanence criteria for student athletes are extended and made more flexible.

Partial enrollment: Students are allowed partial enrollment in certain semesters to reduce the academic load when they have intensive athletic commitments, which helps them avoid overload and focus on their athletic performance when necessary.

Free semesters: Semesters off or planned rest periods are offered to allow student athletes to concentrate exclusively on their training and competitions during certain periods of the year.

Separate academic group: Students are offered the option to be part of a separate academic group composed exclusively of dual-career student athletes, providing them with an additional supportive environment and the opportunity to share similar experiences.

Academic tutoring: A specialized academic tutor is assigned to provide individualized support to student athletes, helping them to manage their workload, develop effective study strategies and maintain a good balance between sport and studies. The tutor also acts as a liaison between the student athlete and the faculty of the different subjects of their degree.

Career advice: Career advice is offered to help them explore career options that align with their interests, skills and athletic goals, preparing them for a successful transition into the workforce once they complete their studies.

SPORTS BENEFITS

Free use of sports facilities: Student athletes are offered free access to the university's sports facilities or to those facilities with an agreement with the university, which allows them to train and practice at no additional cost. This helps them to be able to train in the same place where they study, without the need for long journeys.

Private use of sports facilities: Student athletes are given the opportunity to use the university's sports facilities - or those facilities with an agreement with the university - at exclusive times reserved for elite athletes, which provides them with a more private environment to focus on their training needs.

Reservation of places for sports courses: Specific places are reserved in sports-related courses for dual-career student athletes, allowing them to access specialized and complementary training for their sports career.

Extra credits for participation in university sport events: ECTS credits are awarded for active participation in university sporting events, thus recognizing the time and effort dedicated to representing the institution in sporting competitions.

DC tutoring: Specialized tutoring is offered for the administrative management of dual career student athletes, providing them with individualized support to balance their athletic and academic commitments. This includes advice on time management and the development of skills for success in both fields.

HEALTH-RELATED BENEFITS

General medical services: Student athletes have access to general medical services within the educational institution, allowing them to receive basic medical care to keep their health in optimal condition and treat any ailments or illnesses they may have.

Mental health support: Student athletes are provided with mental health support and resources, such as psychological counseling sessions or access to support groups, to help them manage stress, competitive pressure and other emotional challenges related to their dual career.

Physiotherapy: Student athletes have access to physiotherapy services for the treatment and prevention of sports injuries, as well as for post-injury rehabilitation. This allows them to stay in optimal physical shape and recover more quickly from any physical setbacks.

Nutritionist: Student athletes can receive personalized nutritional advice from a nutritionist to optimize their sports performance and support their overall health. A proper diet is critical to success in sports and academics, and nutritional counseling can help them achieve their goals.

Testing (physiology, biomechanics, performance): Student athletes have the opportunity to undergo various fitness assessment tests (physiology, biomechanics or performance) to assess their fitness and obtain specific information that can help them improve their training and athletic performance.

Specialized PE teachers: Student athletes receive instruction from specialized physical education teachers who can provide them with advanced knowledge and techniques in specific areas of sports interest.

OTHER BENEFITS

Housing: Student athletes are provided with information on suitable accommodation or are provided with accommodation either in student residences, university apartments or private accommodations, ensuring that they have a safe and comfortable place to live during their period of study and training.

Discounts on meals: Student athletes have access to special discounts at university dining halls or restaurants.

Adapted catering service: An adapted catering service is offered to meet the specific needs of student athletes, providing food options and flexible schedules to meet their demanding training and competition schedules.

Scholarships: Scholarships are offered to student athletes to help cover costs related to their studies, such as tuition, books, materials or general expenses.

Extra points in Erasmus evaluation: Student athletes are given extra points in the evaluation to participate in mobility programs such as Erasmus+, thus increasing their chances of benefiting from mobility programs.
